# Imaging tau pathology in Alzheimer’s disease with positron emission tomography: lessons learned from imaging-neuropathology validation studies

**DOI:** 10.1186/s13024-022-00543-x

**Published:** 2022-06-03

**Authors:** Alexis Moscoso, Melissa C. Wren, Tammaryn Lashley, Erik Arstad, Melissa E. Murray, Nick C. Fox, Kerstin Sander, Michael Schöll

**Affiliations:** 1 Department of Psychiatry and Neurochemistry, Institute of Neuroscience and Physiology, The Sahlgrenska Academy, University of Gothenburg, Gothenburg, Sweden; 2 Wallenberg Centre for Molecular and Translational Medicine, University of Gothenburg, Gothenburg, Sweden; 3 Department of Imaging, Centre for Radiopharmaceutical Chemistry, University College London, London, UK; 4 Department of Chemistry, University College London, London, UK; 5 Queen Square Brain Bank for Neurological Disorders, Department of Clinical and Movement Neurosciences, Queen Square Institute of Neurology, University College London, London, UK; 6 Department of Neurodegenerative Disease, Queen Square Institute of Neurology, University College London, London, UK; 7 Department of Neuroscience, Mayo Clinic, Jacksonville, FL USA; 8 Dementia Research Centre, Department of Neurodegenerative Disease, Queen Square Institute of Neurology, University College London, London, UK; 9 UK Dementia Research Institute, Queen Square Institute of Neurology, University College London, London, UK; 10 Department of Clinical Physiology, Sahlgrenska University Hospital, Röda stråket 10B, 413 45 Gothenburg, Sweden

Though the presence of both amyloid-β (Aβ) plaques and tau neurofibrillary tangles is necessary for neuropathologic diagnosis of Alzheimer’s disease (AD), it is now widely recognized that tau burden correlates more strongly with neurodegeneration and cognitive impairment in life than the development of Aβ plaques [1]. Recent developments of tau-sensitive radiotracers for imaging with positron emission tomography (PET) have, for the first time, enabled visualisation, mapping, and quantification of inclusions of aggregated, paired helical filament (PHF) tau associated with AD in the living brain [2]. In-depth characterisation of tau PET tracers, and in particular comparison of antemortem PET readings with postmortem neuropathologic findings, were of paramount importance to understand the clinical potential and limitations of the new imaging tools. In the case of [^18^F]flortaucipir, the most widely used tau PET ligand, these cross-validation studies, together with autoradiography evaluations, provided information about the specificity of this tracer to PHF-tau in AD but also revealed substantial undesired (off-target) binding and limited ability to detect PHF-tau at the earliest Braak stages [3–7]. The combined data subsequently underpinned the implementation of an effective method for the clinical interpretation of [^18^F]flortaucipir PET scans [3]. Ultimately, these efforts have led to the approval of [^18^F]flortaucipir by the US Food and Drug Administration (FDA) as the first PET radiopharmaceutical indicated to ‘estimate the density and distribution of aggregated neurofibrillary tangles in patients with cognitive impairment who are being evaluated for AD (Tauvid prescribing information, https://www.accessdata.fda.gov/drugsatfda_docs/label/2020/212123s000lbl.pdf).

## Inconsistent findings?

In the early days of [^18^F]flortaucipir PET, several research groups identified a sequence of regional binding profiles that approximated the histopathologic stages of tau progression as originally proposed by Heiko and Eva Braak [8], suggesting that [^18^F]flortaucipir PET was suitable for ‘in vivo Braak staging’ [9]. These results were reinforced by studies using autoradiography techniques, which demonstrated correlation of [^18^F]flortaucipir binding with PHF-tau density even in tissue from individuals at the earliest Braak stages [6]. However, despite these promising results, subsequent imaging-neuropathology validation studies showed that antemortem [^18^F]flortaucipir PET scans failed to yield sufficient signal-to-noise ratio in patients who were found to be at early Braak stages (I-IV) in postmortem neuropathologic examinations [3–5]. This suggests that [^18^F]flortaucipir PET might not be suitable—at the individual level—to perform the full range of in vivo staging that would parallel actual Braak stages, even though a spatial distribution of tau may be present across the brain. The apparent mismatch between the two modalities raises important questions: what do PET-derived ‘Braak stages’ actually reflect? How can these apparently discordant results be explained? The purpose of this perspective is to explain this apparent inconsistency, elaborating on the conceptual implications for current and future tau PET research.

## Tau visualisation techniques

To gain better insight into this problem, it is necessary to consider development of neuropathology-derived Braak staging, as well as the differential features of immunohistochemical staining and PET imaging. In their seminal study [8], Braak & Braak oversampled 40 brains to investigate 12–14 tissue blocks. Through their meticulous neurohistologic evaluation using tau immunohistochemistry, they systematically evaluated topographic involvement of PHF-tau throughout the brain. Their semi-quantitative assessment of PHF-tau in neurofibrillary tangles and neuropil threads was used to inform a more focused set of 4 tissue blocks recommended for use in their staging criteria. Thus, immunohistochemical evaluation allows for individual lesions to be visualised, but to offset time-intensive evaluation only an approximate picture of PHF-tau topographic involvement throughout the brain (Braak staging) is assessed by neuropathologists.

In contrast, PET imaging is fully quantitative and allows for a more accurate spatial assessment; however, due to non-specific binding and the limited spatial resolution of PET scanners, this technique requires not only presence, but sufficient density and spatial extent of tau inclusions in a brain region to generate sufficient signal-to-noise ratio. Whereas, immunohistochemical staining allows for visualisation of tau inclusions in densities that are too low to be detected with PET imaging. Autoradiography—nuclear imaging in tissue sections—bridges immunohistochemical staining and in vivo imaging by providing both high sensitivity and resolution (pixels in the μm range) as well as quantification. Yet, similar to neuropathologic examinations, the topography of the target distribution in autoradiography can only be extrapolated by using small brain samples.

It should be noted that the nature of the ligands used in immunohistochemical staging (phospho-tau specific antibodies) and PET (small molecules, e.g., [^18^F]flortaucipir) give rise to differential binding profiles on the molecular level (independent of resolution). However, macroscopic binding patterns of the two types of the ligands overlap in AD tissue. Both antibodies and PET tracers bind to neurofibrillary tangles, but neuropil threads and pre-tangles may have a different binding profile [10].

## Dynamics of tau accumulation

The way tau accumulates in the brain is also relevant to understand differences between immunohistochemical staining and PET. Tau accumulation in AD follows a hierarchical spatial pattern of tau lesions that represents the basis for neuropathologic staging. Whilst Braak stages describe the topographic spread of lesions in the brain, it is hypothesized that tau accumulation is a function of spreading and replication of tau seeds. According to a recent study, the kinetics of tau accumulation were found to change from predominantly spreading during early Braak stages (I-III) to predominantly local replication for Braak stages IV-VI [11]. Interestingly, this kinetic switch approximately coincides with the first manifestation of an elevated signal in antemortem [^18^F]flortaucipir PET scans, which may indicate that at this Braak stage the threshold density for detection is surpassed.

## Tau topography vs tau burden

We hypothesize that the mismatch between neuropathologic Braak- and [^18^F]flortaucipir PET-derived tau stages can be explained by the differential sensitivity of the tau visualisation techniques in conjunction with the above-described switch in the kinetics of tau accumulation. In this theoretical framework, tau neuropathology, as detected at neuropathologic examination with immunohistochemical staining, would initially appear across regions corresponding to Braak areas I to IV. Given that spreading predominates at this stage, the density of PHF-tau is too low to produce optimal signal-to-noise ratio sufficient for detection on a [^18^F]flortaucipir PET scan. Later, when tau pathology appears in Braak areas V-VI but is still not detectable with [^18^F]flortaucipir PET, the density of PHF-tau would begin to exceed the detectability threshold in the previous Braak regions, reproducing the stereotypical accumulation from Braak areas I to IV. Finally, PHF-tau density continues increasing in Braak areas V-VI, eventually resulting in [^18^F]flortaucipir PET signal elevations within these regions. This temporal evolution of events implies that, in brains that follow the stereotypic tau accumulation pattern, [^18^F]flortaucipir PET-derived ‘Braak staging’ would not reflect the present but, instead, a past neuropathologic Braak stage. Therefore, consideration of this temporal delay when interpreting PET scans should advise caution, as the absence of an elevated signal in a given brain region does not exclude the possibility that tau has readily spread therein. At the current stage, this hypothetical model needs further validation in imaging-neuropathology studies including more individuals with early-stage tau pathology (Braak stages III-IV), allowing for a definite, reliable assessment of the temporal sequence predicted by our model.

Despite the limited ability of [^18^F]flortaucipir PET to resolve tau inclusions at early disease stages, its unique strength—the ability to quantitatively assess tau burden in a continuous rather than dichotomous (presence or absence of elevated signal) manner—should be emphasised. Importantly, tau *burden* rather than propagation has been found to correlate with neuronal injury and severity of clinical symptoms [1]. Therefore, the quantification of the density of tau inclusions might add relevant information that is not captured by traditional Braak staging. Changes of continuous tau density over time, as measured by tau PET, may provide more valuable information about disease progression, and potentially allow assessment of tau modifying therapeutic interventions.

## The promise of next-generation tau tracers

**Fig. 1:**
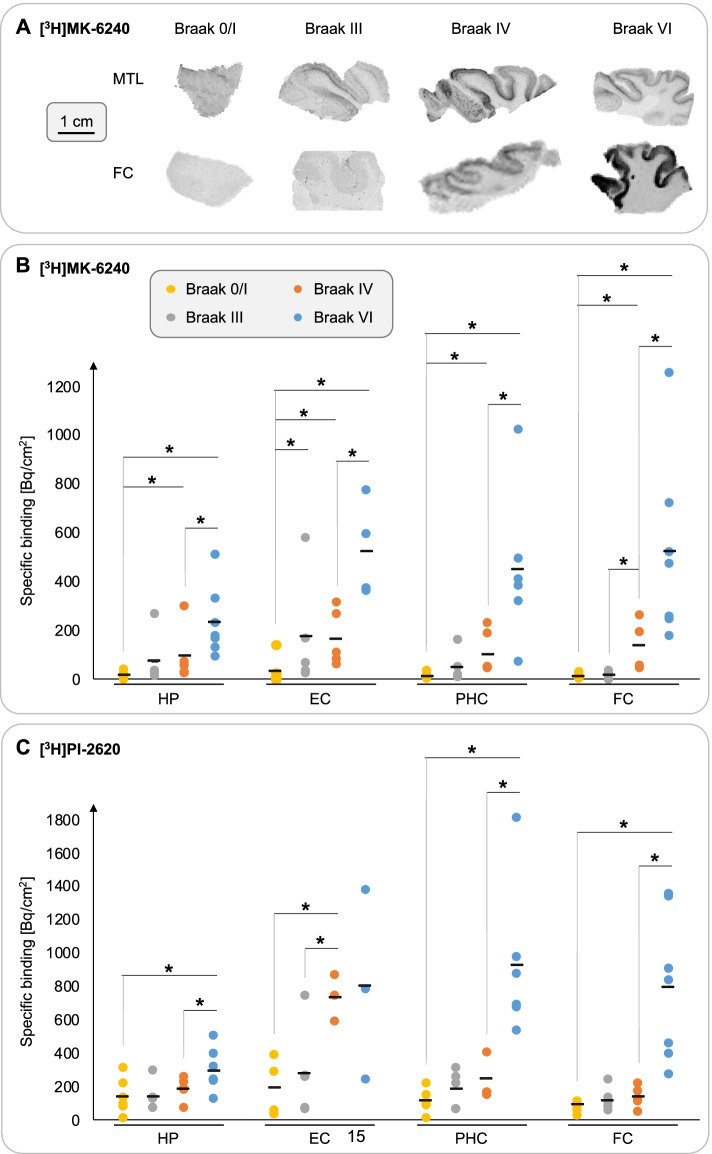
Next-generation tau tracer binding to human postmortem brain tissue. **A** Representative sections derived from the medial temporal lobe (MTL) and frontal cortex (FC; Brodmann area 9) showing increased [^3^H]MK-6240 binding, which was most abundant in cortical layer V during early Braak stages III/IV; additional binding to cortical layer III was observed in tissue from AD cases (Braak stage VI). **B/C** Specific [^3^H]MK-6240 and [^3^H]PI-2620 binding to directly adjacent tissue sections from a cohort of 24 cases (Braak 0/I: *n* = 7; Braak III: *n* = 5; Braak IV: *n* = 5; Braak VI: *n* = 7) showing pronounced increase in tracer binding with advancing Braak stage. A previously published protocol was used for the autoradiography experiments [7]. Abbreviations: HP: Hippocampus; EC: Entorhinal cortex; PHC: Parahippocampal cortex. *p < 0.05

The limitations of [^18^F]flortaucipir sparked the development of a new generation of PET ligands. Radiotracers such as [^18^F]PI-2620, [^18^F]RO-948, [^18^F]GTP-1 and [^18^F]MK-6240 have improved specificity for PHF-tau and are therefore suited to depict tau associated with AD [2, 7]. Here, we provide novel data suggesting that the structurally distinct next-generation candidate tracers [^3^H]PI-2620 and [^3^H]MK-6240 display significantly increased binding to PHF-tau in postmortem brain tissue from cases characterised to have reached Braak stages III and IV (Fig. 1, see [7] for a detailed description of autoradiographic experimental methods). Remarkably, group-level tracer binding in the parahippocampal cortex almost doubled in cases with Braak stage III and IV. Furthermore, [^18^F]MK-6240 displays high affinity to PHF-tau (in the subnanomolar concentration range), which may have practical implications for the detection of tau inclusions during the early stages of AD and quantification of subtle changes in tau burden over time [7]. Together, these observations hold promise for next-generation PET ligands as tools for in vivo staging of tau pathology, yet the impact of specific limitations of these tracers, such as off-target binding in the meninges, still needs to be investigated. Imaging-neuropathology studies will definitively establish whether next-generation tracers will provide sufficient signal-to-noise ratio to detect early tau inclusions in AD.

## Lessons learned

To date, we believe that the tau PET research community has prioritized the concept of topographic spreading of tau over the concept of tau burden quantification, even though lessons learned from imaging-neuropathology correlation studies indicate that the former reflects a rather incomplete picture of disease progression. Taking advantage of the unique features of PET as an imaging modality will allow strategic development for disease staging that takes tau distribution patterns as well as burden into account. New generations of tau PET tracers may ultimately provide the necessary sensitivity to visualise early tau pathology in AD and perform in vivo tau staging. However, we should not assume this ability solely based on positive findings from autoradiography and in vivo PET studies: this can only be finally demonstrated with imaging-neuropathology correlation studies.

## Abbreviations

Aβ: Amyloid beta
AD: Alzheimer’s disease
FC: Frontal cortex
FDA: US Food and Drug Administration
MTL: Medial temporal lobe
PET: Positron emission tomography
PHF: Paired helical filament
